# Genomic prediction based on data from three layer lines: a comparison between linear methods

**DOI:** 10.1186/s12711-014-0057-5

**Published:** 2014-10-01

**Authors:** Mario PL Calus, Heyun Huang, Addie Vereijken, Jeroen Visscher, Jan ten Napel, Jack J Windig

**Affiliations:** Animal Breeding and Genomics Centre, Wageningen UR Livestock Research, P.O. Box 338, 6700 AH Wageningen, The Netherlands; Hendrix Genetics Research, Technology & Services B.V., P.O. Box 114, 5830 AC Boxmeer, The Netherlands; ISA, Institut de Sélection Animale, P.O. Box 114, 5830 AC Boxmeer, The Netherlands

## Abstract

**Background:**

The prediction accuracy of several linear genomic prediction models, which have previously been used for within-line genomic prediction, was evaluated for multi-line genomic prediction.

**Methods:**

Compared to a conventional BLUP (best linear unbiased prediction) model using pedigree data, we evaluated the following genomic prediction models: genome-enabled BLUP (GBLUP), ridge regression BLUP (RRBLUP), principal component analysis followed by ridge regression (RRPCA), BayesC and Bayesian stochastic search variable selection. Prediction accuracy was measured as the correlation between predicted breeding values and observed phenotypes divided by the square root of the heritability. The data used concerned laying hens with phenotypes for number of eggs in the first production period and known genotypes. The hens were from two closely-related brown layer lines (B1 and B2), and a third distantly-related white layer line (W1). Lines had 1004 to 1023 training animals and 238 to 240 validation animals. Training datasets consisted of animals of either single lines, or a combination of two or all three lines, and had 30 508 to 45 974 segregating single nucleotide polymorphisms.

**Results:**

Genomic prediction models yielded 0.13 to 0.16 higher accuracies than pedigree-based BLUP. When excluding the line itself from the training dataset, genomic predictions were generally inaccurate. Use of multiple lines marginally improved prediction accuracy for B2 but did not affect or slightly decreased prediction accuracy for B1 and W1. Differences between models were generally small except for RRPCA which gave considerably higher accuracies for B2. Correlations between genomic predictions from different methods were higher than 0.96 for W1 and higher than 0.88 for B1 and B2. The greater differences between methods for B1 and B2 were probably due to the lower accuracy of predictions for B1 (~0.45) and B2 (~0.40) compared to W1 (~0.76).

**Conclusions:**

Multi-line genomic prediction did not affect or slightly improved prediction accuracy for closely-related lines. For distantly-related lines, multi-line genomic prediction yielded similar or slightly lower accuracies than single-line genomic prediction. Bayesian variable selection and GBLUP generally gave similar accuracies. Overall, RRPCA yielded the greatest accuracies for two lines, suggesting that using PCA helps to alleviate the “*n* ≪ *p*” problem in genomic prediction.

**Electronic supplementary material:**

The online version of this article (doi:10.1186/s12711-014-0057-5) contains supplementary material, which is available to authorized users.

## Background

In recent years, genomic prediction has been adopted in many breeding programs for the main livestock species, to enable genomic selection [1] instead of traditional selection based on performance of selection candidates or close relatives such as sibs or offspring. At the same time, much research effort has been geared towards developing models for genomic prediction (for a review, see [2]). Most of these are linear models, which can roughly be divided into three groups. One group assumes that all SNPs (single nucleotide polymorphisms) contribute equally to genetic variance, and therefore apply equal shrinkage to the effects of each SNP. This group includes models such as genomic best linear unbiased prediction (GBLUP) [3], and random regression or ridge regression-BLUP (RRBLUP) [4], which are known to be equivalent [5] and effectively use genome-wide relationships that are computed from the SNPs. A second group of models avoids the use of shrinkage by linear dimensionality reduction of the SNP genotypes. This group includes principal component regression e.g. [6]. A third group of models includes methods that allow for different contributions to genetic variance across SNPs by differential shrinkage e.g. BayesA and BayesB [1], BayesC [7], and Bayesian stochastic search variable selection [8,9].

Many studies have compared the performance of different linear genomic prediction models (for a review, see [2]). Most of these comparisons used data of a single breed or line. Within a single breed or line, linkage disequilibrium (LD) between QTL (quantitative trait loci) and SNPs may extend across a relatively large distance, and therefore a QTL is expected to be in LD with several surrounding SNPs. In such situations, genomic prediction models can apply various strategies to capture the QTL effects. They can, for instance, try to put most of the effect on the SNP that has the highest LD with the QTL, or distribute the QTL effect across multiple surrounding SNPs. The first strategy may be more easily achieved with a model that allows for differential shrinkage, while the second strategy may be more easily achieved with a GBLUP or RRBLUP type of model. Despite these differences in strategies, in general the different models yield very similar predictive abilities, which suggests that for within-breed or within-line selection, the strategy that the model uses has generally limited impact on the results.

For applications of across-breed or -line genomic prediction, it is important that the LD between SNPs and QTL is the same across those breeds or lines [10]. With increasing genetic distance between breeds or lines, a higher SNP density is required to achieve the same LD between SNP and QTL in those breeds or lines [11,12]. This implies that SNP density is an important factor for the accuracy of across-breed prediction [10]. Thus, the number of SNPs that are useful for genomic prediction per QTL is expected to be smaller for across-breed or line applications compared to single-breed or line applications. As a consequence, with increasing distance between breeds or lines, the differential shrinkage models are expected to have a higher predictive ability than their GBLUP type counterparts, which is supported by a few empirical studies that reported slightly higher accuracies for differential shrinkage models applied to multi-breed training datasets [13,14]. However, when QTL effects differ between lines or breeds, the benefit of using differential shrinkage models may be very small.

The objective of our study was to investigate whether genomic data across lines improves the accuracy of genomic predictions per line, and whether such improvement depends on the linear model used. Specifically, we tested the hypothesis that use of differential shrinkage models is more beneficial when lines are genetically further apart. We used data from three lines of layer chickens, including two closely-related lines of brown layers and one distantly-related line of white layers.

## Methods

### Dataset

To evaluate the usefulness of data from different populations for genomic prediction, three different pure-bred lines of layer chickens were analysed. The brown layer lines B1 and B2 were more closely related to each other than to the white line (W1), albeit that lines B1 and B2 were separated for at least 25 years. All three lines have been selected for egg production. With this data, we were able to investigate the influence of the relatedness of lines on the accuracy of genomic prediction. The trait analysed was number of eggs in the first production period.

A total of 3753 female birds with phenotypes were genotyped with the chicken Illumina Infinium iSelect Beadchip which contains 57 636 SNPs. Edits on the genotype data were performed for the three lines simultaneously. Those edits comprised removing SNPs with a call rate below 95%, a minor allele frequency (MAF) below 2%, that had no homozygous genotypes, or that had a Chi^2^ test for deviation from Hardy-Weinberg equilibrium greater than 600. After these edits, 45 974 SNPs remained. In total, 1263 birds were genotyped in line B1, 1246 in line B2 and 1244 in line W1. Differences between genotypes of birds in different lines were evaluated by comparing allele frequencies between lines. In addition, the Euclidian distances between genotypes of all pairs of birds were computed as $\sqrt{{\displaystyle {\sum}_{i=1}^n{\left({x}_{ij}-{x}_{ik}\right)}^2}}$, where *x*_*ij*_ and *x*_*ik*_ are the genotypes of animals *j* and *k*, respectively, on SNP *i*.

To evaluate the accuracy of genomic prediction, the data was split into training and validation datasets. The validation datasets consisted of the youngest generation of birds, comprising 238 to 240 birds for the three lines. The phenotypes of these birds were set to missing and their breeding values were predicted using the training dataset. Phenotypes were pre-corrected for fixed effects of hatch week. The accuracy of the estimated breeding values (EBV) was computed as the correlation coefficient between the EBV and the observed phenotypes of the validation animals, which was a single own performance record, divided by the square root of the heritability of the trait. Heritabilities from routine genetic evaluations were used, i.e. 0.41 for lines B1 and B2 and 0.51 for line W1. Standard errors of the prediction accuracies were approximated using the expected sampling variance of an estimated correlation $\left(\widehat{\rho}\right)$, e.g. [15], as $\sqrt{\frac{1-{\widehat{\rho}}^2}{N-2}}/\sqrt{h^2}$. Bias of the genomic predictions was assessed by evaluating the coefficient of the regression of phenotypes on EBV of the validation animals. Standard errors of those regression coefficients were computed using bootstrapping with the R-package “boot” [16]. The bootstrapping procedure involved computing regression coefficients for 10 000 bootstrap samples of the validation animals. Standard errors were computed as the standard deviation of those 10 000 estimated regression coefficients. Using those standard errors $\left(S{E}_{b_1}\right)$, the regression coefficients (*b*_1_) were considered to be not significantly different from the expected value of 1 when $\left|{b}_1-1\right|<2\times S{E}_{b_1}$ [17].

For each line, seven training datasets were used to evaluate the accuracy of genomic prediction. As a base, the training dataset consisting of birds of its own line was used. To evaluate across-line genomic prediction, training datasets that included one of the other two lines were evaluated. To evaluate whether genomic prediction can benefit from data from other lines, three training datasets consisting of birds of two of the three lines were composed, as well as a training dataset that included all birds from all three lines. Training datasets with one, two and three lines, included slightly more than 1000, 2000 and 3000 animals, respectively (Table 1). Some of the retained SNPs segregated only in one or two lines, and therefore were not used in some training datasets. Across training datasets, the number of segregating SNPs ranged from 30 508 to 45 974 (Table 1).

**Table 1:** **Number of animals and number of segregating SNPs for each training dataset**

**Training**	**Number of animals**	**Number of segregating SNPs**
B1	1023	38 310
B2	1008	37 729
W1	1004	30 508
B1 + B2	2031	40 953
B1 + W1	2027	45 241
B2 + W1	2012	44 913
B1 + B2 + W1	3035	45 974

### Linear models for genomic prediction

Eight methods for genomic prediction were evaluated and compared to a pedigree-based BLUP model. These methods included three different implementations of GBLUP, two implementations of RRBLUP, RRPCA, Bayesian stochastic search variable selection (BSSVS) and BayesC. In the following, we start with a general description of linear genomic prediction models, followed by a short description of each individual model.

Genomic prediction is aimed at predicting the phenotype *y* of an animal using its *p* SNP genotypes **x** by uncovering the implicit mapping function *y* = *f* (**x**). Linear models assume that the mapping function is linear by *f *(**x**) = **w**^*t*^**x**. The vector **w** contains the linear weights imposed on SNP genotypes, which effectively are the marker effects. As a learning problem, the marker effects are estimated from a training dataset consisting of *n* animals whose genotypes and phenotypes are characterized by (**x**_i_, *y*_*i*_), for animal *i* = 1, 2, …, *n*. The marker effects are estimated by minimizing the prediction error (*e*_*i*_) computed from the observed phenotype value *y*_*i*_ and estimated phenotype value **w**^*t*^**x**_i_ within the training dataset as:

1 $$ L\left(\mathbf{w}\right)={\displaystyle {\sum}_{i=1}^n{e}_i^2}\equiv {\displaystyle {\sum}_{i=1}^n{\left({y}_i-{\mathbf{w}}^t{\mathbf{x}}_i\right)}^2.} $$

Minimization of the loss function *L* in Equation (1) with regard to **w** results in the following estimate [2,18]:

2 $$ {\mathbf{w}}^{*}={\left({\mathbf{X}}^t\mathbf{X}\right)}^{-1}{\mathbf{X}}^t\mathbf{y}, $$

where matrix **X** contains the genotypes of the training animals and **y** is a column vector composed of all phenotypes.

### The need for regularization

One of the major problems of linear regression applied for genomic prediction is the over-fitting phenomenon caused by the fact that the number of training animals is generally much smaller than the number of genotypes (*n* < < *p*), which is also known as the small sample-to-size (SSS) problem in general machine learning theory [19,20]. One straightforward drawback is that the solution to **w** depends on a non-invertible matrix, **X**^*t*^**X**, which is the so-called ‘ill-posed’ problem [21]. This problem is more severe when higher-density SNP panels are used, which are expected to convey more accurate information on the animals. Another well-known disadvantage of linear regression is that it is too flexible in cases with an enormous number of (highly) correlated covariates that are used for prediction [22,23]. To overcome this problem, a regularization parameter is added to the model, which in the case of genomic prediction includes the variances attributed to each SNP, e.g. [1]. Thereby, this regularization term can act as an important carrier to incorporate prior information into the regression model [24–26]. In other words, it may be helpful to select SNPs that are *a priori* known to be important, instead of completely learning the weights of each SNP from the regression model.

To overcome the limited size of the training dataset, the structures of linear weights **w** are incorporated into the regression framework. There are three main approaches to perform shrinkage on the marker effects [2]: (1) penalizing **w**, (2) applying differential shrinkage to **w** using probabilistic modelling, and (3) reducing the length of **w**. [6,27]. In this section, several state-of-the-art regression models from these three categories are described, which will be adopted for the multi-line genomic prediction.

### Ridge regression with best linear unbiased predictor and GBLUP

Ridge regression penalizes the sum of squares of **w** with the aim of controlling the arbitrary scale of regression coefficients, which makes it possible to alleviate over-fitting on the training dataset. In concrete terms, the linear regression in Equation (1) is modified as follows:

3 $$ L\left(\mathbf{w}\right)={\left|\left|\mathbf{y}-\mathbf{X}\mathbf{w}\right|\right|}^2+\alpha {\left|\left|\mathbf{w}\right|\right|}^2, $$

where ||**w**||^2^ is the 2-norm of the vector **w**, which is the regularization term and *α* controls the trade-offs between the prediction error and model complexity. When *α* goes to 0, this model reduces to Equation (1). Using Equation 3, the solution becomes:

4 $$ {\mathbf{w}}^{*}={\left({\mathbf{X}}^t\mathbf{X}+\alpha \mathbf{I}\right)}^{-1}{\mathbf{X}}^t\mathbf{y}. $$

The BLUP models assume that *α*, computed from the error variance ${\sigma}_e^2$ and ${\sigma}_w^2$, which are SNP variances that are assumed to be the same for all SNPs is equal to:

$$ \alpha =\frac{\sigma_e^2}{\sigma_w^2}. $$

Inserting this definition of *α* into the predictor of Equation (4) results in the well-known RRBLUP model [1,4]. In our study, RBLUP was solved using the preconditioned conjugated gradient method implemented in the software package MiXBLUP [28]. In this implementation, genotypes are centred and scaled before being included in the model. Results obtained with an implementation using Gauss-Seidel, similar to Legarra and Misztal [29], were very similar and are therefore not presented.

Method GBLUP has been shown to be mathematically equivalent to the RRBLUP model [5]. In our study, we applied three different implementations of GBLUP. The first, hereafter referred to as GBLUP_VR, used a genomic relationship matrix (**G**) that is computed as described by VanRaden [30]. The **G** matrix was computed once, including all three lines, and used for all training datasets. The second, hereafter referred to as GBLUP_%id, computed **G** as the fraction of SNP alleles identical between two individuals, where loci with identical homozygotes were scored as 1, opposite homozygotes as 0, and all others as 0.5. The third computed **G** as the excess of identical homozygotes based on expected homozygosity: ([O(H_identical_) – O(H_opposite_)] – E(H))/(*p* – E(H)), where E(H) is the expected number of homozygous SNP genotypes based on allele frequencies obtained from the whole population, O(H_identical_) is the observed number of identical homozygous SNP genotypes in the two individuals, and O(H_opposite_) is the observed number of opposing homozygous SNP genotypes in the two individuals. These estimates were obtained from PLINK, using the “–het” command. Preliminary analyses showed that GBLUP_VR and the third GBLUP model gave very similar results (correlation between EBV > 0.996), so results of the third GBLUP model were not presented.

### Bayesian stochastic search variable selection and BayesC

Another commonly used class of genomic prediction models are the variable selection models. These models perform a regression on SNP genotypes similar to RRBLUP, but the variance assigned to each SNP $\left({\sigma}_w^2\right)$ is estimated in the model and may have a different value for each SNP. Specifically, both BayesC and Bayes stochastic search variable selection (BSSVS) determine in each iteration of the Gibbs chain whether a particular SNP has a large effect on the phenotypes or not. If the SNP does not have a large effect, then BayesC effectively sets its effect to 0 in that iteration of the chain [7], while BSSVS estimates and assigns a small effect to the SNP [8,9]. In this way, variable selection models are able to better fit traits that are (at least partly) underpinned by QTL of large effect [31,32]. Details on the implementation of both models are presented elsewhere [33]. Based on past experience, the parameter π that specifies the proportion of loci that does not have a large effect, was set to 0.9 for BayesC and to 0.999 for BSSVS.

### Principal component regression

The underlying idea of both penalized regression and the above described Bayesian models is to limit the degrees of freedom to estimate the marker effects **w**. Principal component analysis (PCA) is compatible with most regression models since it enables pre-processing of the data that are used in the subsequent regression models. The low-dimensional representation of the genotype data that results from the use of PCA, implies that computational costs are considerably lower than for other models that explicitly include genotypes of all SNPs. To the best of our knowledge, the use of regression on principal components (PC) of genotypes for genomic prediction has only been studied in the context of a single line or breed [27,34–36] but not for genomic prediction based on multiple lines or breeds. To investigate the potential of principal component regression for multi-line genomic prediction, an in-depth analysis of PC of the SNP genotypes will be presented.

PCA is one of the most widely-used dimensionality reduction algorithms. Its target is to combine a large number of correlated covariates into a limited number of PC that explain a maximum amount of variance in the data. In the context of genomic prediction, this means that the information of a number of SNPs that are correlated are combined into PC, which are then included in the genomic prediction model based on decreasing the proportion of the total variance explained. As a result, SNPs with low MAF may not be included in the prediction due to their expected lack of relevant information.

Combining the genotypes of all SNPs could be done by using a linear projection **z** = **Tx**, where the matrix **T** is the projection matrix with dimension *d* × *p* (*d* < *p*), where *p* is the number of animals, *d* is the number of PC retained (i.e. those with the largest eigenvalues), and *d* is the low-dimensional representation of **x**. The objective function is to maximize the amount of the total genotypic variance captured by the reduced vector **z** over the entire training dataset:

$$ \mathrm{maximize}:\  trace\left(\mathbf{T}\mathbf{X}{\mathbf{X}}^{\mathrm{t}}{\mathbf{T}}^{\mathrm{t}}\right) $$

$$ \mathrm{Subject}\ \mathrm{t}\mathrm{o}: trace\left(\mathbf{T}{\mathbf{T}}^{\mathrm{t}}\right)=1, $$

where the operator *trace* refers to the sum of the diagonal elements of the denoted matrix and matrix **X** contains only the SNP genotypes of the training animals. The solution of the projection matrix **T** is the first *d* eigenvectors of the covariance matrix of genotypes, **XX**^t^. To complete the regression analysis, the standard regression model as shown in Equation (4) is applied, which is hereafter referred to as RRPCA.

For all training scenarios, PC were derived based on the genotypes of the training animals of all three lines simultaneously. The number of PC used in RRPCA was determined by selecting the minimum number of PC required to explain 97% of the variation in the genotype data. In addition, the prediction accuracy of PCA was evaluated across the whole range of PC, to investigate the potential of PCA, assuming the user is able to determine the most optimal settings a priori.

### Variance components

The variance components used in the models as well as to compute the accuracies were derived from parameters used in routine breeding value estimation procedures that were applied in the breeding program from which the analyzed layer lines originated. When the training dataset contained multiple lines, the average of the variance components across those lines was taken. In the models that explicitly used (RRBLUP) or estimated SNP effects (BSSVS and BayesC), the (prior) SNP variance was computed as the total genetic variance divided by the number of SNPs, because the genotypes were centred and scaled such that they had a variance of 1 for all these models.

### Pedigree-based prediction

To evaluate the benefit of using genomic prediction models over the use of conventional BLUP models that rely on pedigree data, the latter model was also fitted to the training datasets. In this model, the variance components used were the same as those used for the GBLUP model. For each line, 9 to 10 generations of pedigree data were available, with a total of 27 808 to 31 060 animals per line.

## Results

### Differences and similarities between lines

**Figure 1:**
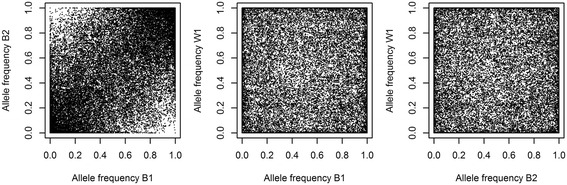
**Pairwise comparisons of allele frequencies in the three layer lines.**

**Figure 2:**
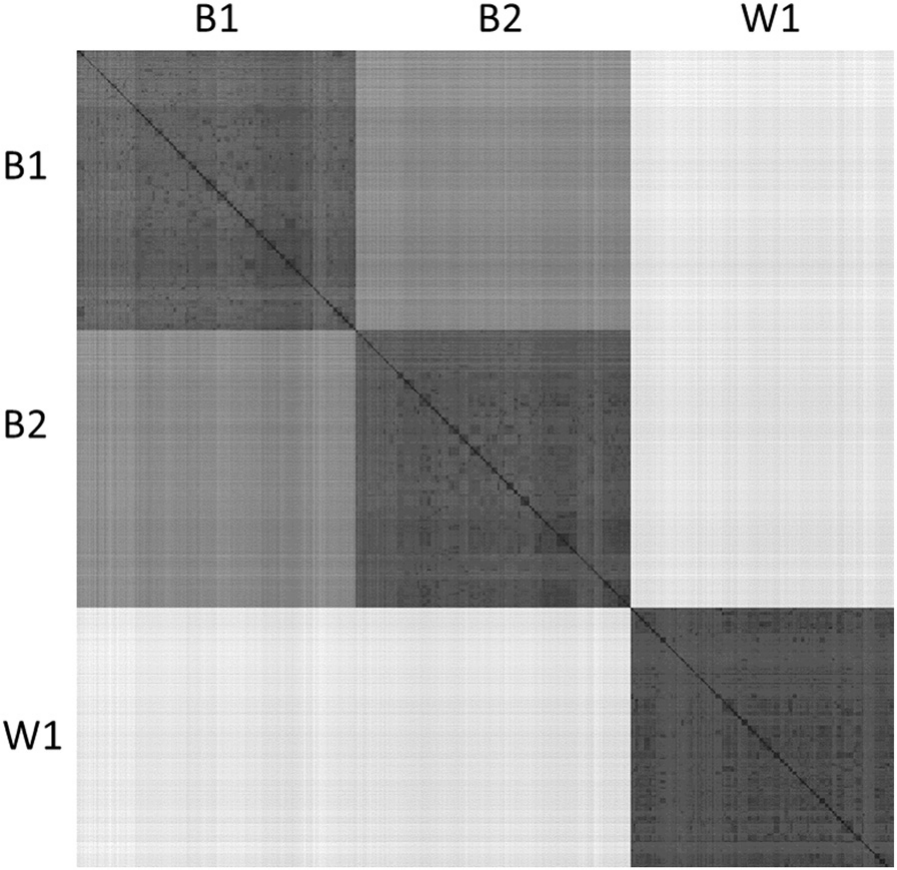
**Visualization of the distance matrix of the multi-line training dataset.** The Euclidean distance is computed as a measure of the distance between any two individuals based on their genotypes. The distance is visualized from very small distances (black) to very large distances (light grey).

Differences between lines were first evaluated by comparing SNP allele frequencies for each pair of lines (Figure 1). The MAF of the SNPs in lines B1 and B2 had a correlation (*r*) of 0.35. Based on this, these two lines were more similar than line B1 versus line W1 (*r* = 0.11) and line B2 versus line W1 (*r* = -0.09). The number of fixed SNPs was 8440 (18.4%) for line B1, 8533 for line B2 (18.6%) and 19 412 (42.2%) for line W1. Of the SNPs that were fixed in line B2, 5404 were fixed for the same allele in line B1, but only 854 in line W1, while 663 alleles were fixed for the same allele in lines W1 and B1. Three SNPs were fixed for opposite alleles in lines B1 and B2, while 1548 and 1492 SNPs were fixed for opposite alleles in line W1, and lines B1 and B2, respectively. The lower relatedness of line W1 to the other two lines, as observed from differences in MAF and fixed alleles, was also reflected by the average Euclidian distance between genotypes of birds of different lines (Figure 2). Within-line, birds of line W1 were on average more related than birds in line B1 and line B2.

### Accuracy of genomic prediction within and across lines

Accuracies of the genomic predictions in the validation data, when using only the line itself in the training dataset, were, on average, 0.13, 0.13 and 0.16 higher than accuracies obtained from the regular BLUP model for lines B1, B2, and W1, respectively (Tables 2, 3 and 4). In the following, results for the genomic prediction models are presented for each line, separately.

**Table 2:** **Accuracy** ^**1**^ **of prediction of seven linear methods in seven training scenarios for line B1**

	**Training dataset**						
**Model**	**B1**	**B2**	**W1**	**B1 + B2**	**B1 + W1**	**B2 + W1**	**B1 + B2 + W1**
BLUP^2^	0.336	-	-	-	-	-	-
GBLUP_VR	0.504	0.285	−0.052	0.494	0.479	0.233	0.476
GBLUP_%id	0.512	0.312	−0.068	0.515	0.489	0.234	0.504
RRBLUP	0.453	0.302	−0.003	0.467	0.438	0.272	0.452
RRPCA	0.447	0.230	0.100	0.439	0.436	0.244	0.432
BSSVS	0.456	0.261	−0.095	0.465	0.436	0.220	0.447
BayesC	0.452	0.266	−0.093	0.466	0.429	0.215	0.447

BLUP: conventional BLUP using a pedigree based relationship matrix; G-BLUP: Genome-enabled Best Linear Unbiased Prediction (G-BLUP); RRBLUP: Ridge Regression BLUP; RRPCA: Ridge Regression with PCA reduction; BayesSSVS: Bayesian Stochastic Search Variable Selection; BayesC; ^1^approximated SE of the accuracies of the genomic prediction models ranged from 0.096-0.102; ^2^for BLUP, only the analysis including the line itself was performed, because there are no pedigree relations between lines.

**Table 3:** **Accuracy** ^**1**^ **of prediction of seven linear methods in seven training scenarios for line B2**

	**Training dataset**						
**Model**	**B1**	**B2**	**W1**	**B1 + B2**	**B1 + W1**	**B2 + W1**	**B1 + B2 + W1**
BLUP^2^	-	0.220	-	-	-	-	-
GBLUP_VR	0.123	0.301	0.123	0.303	0.173	0.332	0.343
GBLUP_%id	0.147	0.329	0.136	0.336	0.198	0.352	0.376
RRBLUP	0.129	0.359	0.142	0.373	0.176	0.369	0.390
RRPCA	0.143	0.448	0.109	0.476	0.185	0.463	0.494
BSSVS	0.118	0.316	0.112	0.327	0.150	0.346	0.356
BayesC	0.111	0.338	0.106	0.318	0.139	0.354	0.357

BLUP: conventional BLUP using a pedigree based relationship matrix; G-BLUP: Genome-enabled Best Linear Unbiased Prediction (G-BLUP); RRBLUP: Ridge Regression BLUP; RRPCA: Ridge Regression with PCA reduction; BayesSSVS: Bayesian Stochastic Search Variable Selection; BayesC; ^1^approximated SE of the accuracies of the genomic prediction models ranged from 0.097-0.102; ^2^for BLUP, only the analysis including the line itself was performed, because there are no pedigree relations between lines.

**Table 4:** **Accuracy** ^**1**^ **of prediction of seven linear methods in seven training scenarios for line W1**

	**Training dataset**						
**Model**	**B1**	**B2**	**W1**	**B1 + B2**	**B1 + W1**	**B2 + W1**	**B1 + B2 + W1**
BLUP^2^	-	-	0.599	-	-	-	-
GBLUP_VR	−0.339	−0.161	0.768	−0.393	0.747	0.764	0.748
GBLUP_%id	−0.342	−0.141	0.764	−0.378	0.747	0.758	0.746
RRBLUP	−0.252	−0.192	0.761	−0.393	0.743	0.762	0.742
RRPCA	−0.247	−0.249	0.775	−0.352	0.748	0.772	0.747
BSSVS	−0.241	−0.212	0.757	−0.382	0.734	0.758	0.742
BayesC	−0.235	−0.224	0.759	−0.355	0.737	0.757	0.739

BLUP: conventional BLUP using a pedigree based relationship matrix; G-BLUP: Genome-enabled Best Linear Unbiased Prediction (G-BLUP); RRBLUP: Ridge Regression BLUP; RRPCA: Ridge Regression with PCA reduction; BayesSSVS: Bayesian Stochastic Search Variable Selection; BayesC; ^1^approximated SE of the accuracies of the genomic prediction models ranged from 0.054-0.064; ^2^for BLUP, only the analysis including the line itself was performed, because there are no pedigree relations between lines.

Across all models, the highest prediction accuracy for line B1 was always observed when the training data only included line B1 or both lines B1 and B2 (Table 2). Differences in accuracies between these two scenarios were generally very small. Across models, using only information of the closely-related line B2 yielded accuracies that ranged from 0.23 to 0.31.

For line B2, all models showed the highest prediction accuracy when the training data included all three lines (Table 3), which, across models, yielded accuracies that were 0.01 to 0.05 higher than the accuracies obtained when only line B2 itself was included in the training dataset. This suggests that multi-line genomic prediction was beneficial for this line. This is supported by the observation that accuracies for B2 were consistently greater than 0.10 when either line B1 or line W1 were used for training.

For line W1, just using W1 animals in the training dataset generally yielded the highest accuracy (Table 4). Adding training animals from lines B1 and B2, resulted in a small drop in accuracy by ~0.02 and 0.01, respectively. Using, only line B1 or B2, or both, for training resulted in negative accuracies for line W1, which ranged from -0.14 to -0.39.

### Comparison of models

The GBLUP model yielded higher prediction accuracies than the other models for line B1 (Table 2). For line B2, the accuracy of RRPCA was considerably higher than that of the other models for all training datasets that included line B2, including RRBLUP (Table 3). This suggests that the PC enable the most relevant information of the genotypes to be conveyed for this line. For line W1, RRPCA also tended to have the highest accuracy, although the differences with the other models were very small (Table 4).

Predicted genomic breeding values obtained from the different models were compared for the training dataset that included all three lines, by computing correlations between the predictions in the validation data (Table 5). These correlations were in general smallest for line B1 and largest for line W1. The same trend was observed for the correlations between the predictions from BLUP and the genomic prediction models, with average correlations of 0.46, 0.52 and 0.76 for lines B1, B2 and W1, respectively.

**Table 5:** **Correlations between genomic breeding values obtained with different models and a training dataset that includes all three lines**

**Line**	**Method**	**GBLUP_VR**	**GBLUP_%id**	**RRBLUP**	**RRPCA**	**BSSVS**	**BayesC**
B1	BLUP	0.37	0.46	0.49	0.48	0.43	0.42
	GBLUP_VR		0.97	0.96	0.88	0.96	0.96
	GBLUP_%id			0.98	0.92	0.97	0.97
	RRBLUP				0.93	0.98	0.98
	RRPCA					0.90	0.90
	BSSVS						1.00
B2	BLUP	0.46	0.54	0.53	0.44	0.51	0.51
	GBLUP_VR		0.98	0.96	0.89	0.96	0.96
	GBLUP_%id			0.97	0.91	0.96	0.96
	RRBLUP				0.93	0.98	0.98
	RRPCA					0.91	0.91
	BSSVS						1.00
W1	BLUP	0.73	0.77	0.78	0.73	0.75	0.75
	GBLUP_VR		0.98	0.98	0.96	0.99	0.99
	GBLUP_%id			1.00	0.97	0.99	0.99
	RRBLUP				0.98	0.99	0.99
	RRPCA					0.97	0.97
	BSSVS						1.00

Correlations between predictions from the same pair of models showed consistent trends across the three lines (Table 5). Despite the equivalence of RRBLUP and GBLUP, correlations between predictions obtained from these two models were as low as 0.96 and were of similar magnitude as correlations with predictions from the Bayesian models. The correlation between predictions from BayesC and BSSVS was equal to 1.00 for all three lines. The genomic breeding values obtained with the RRPCA model deviated most from those of the other models and had the lowest average correlations with the other models i.e. 0.91, 0.91 and 0.97 for lines B1, B2 and W1, respectively.

### Bias of genomic prediction within and across lines

Bias of the predicted breeding values is assessed by regressing phenotypes on the predicted breeding values. The coefficients of those regressions (see Additional file 1: Tables S1, S2 and S3) show that, for lines B1 and W1, the variance of the predicted breeding values was underestimated, i.e. all regression coefficients were greater than 1.0. Generally, the regression coefficients were closest to 1 when the line itself was included in the training dataset. In fact, within each model, regression coefficients tended to be very similar across training datasets that included the line itself. This indicates that adding other lines in the training dataset did not affect the scale of the EBV.

### Analysis of principal components of genotypes within and across lines

**Figure 3:**
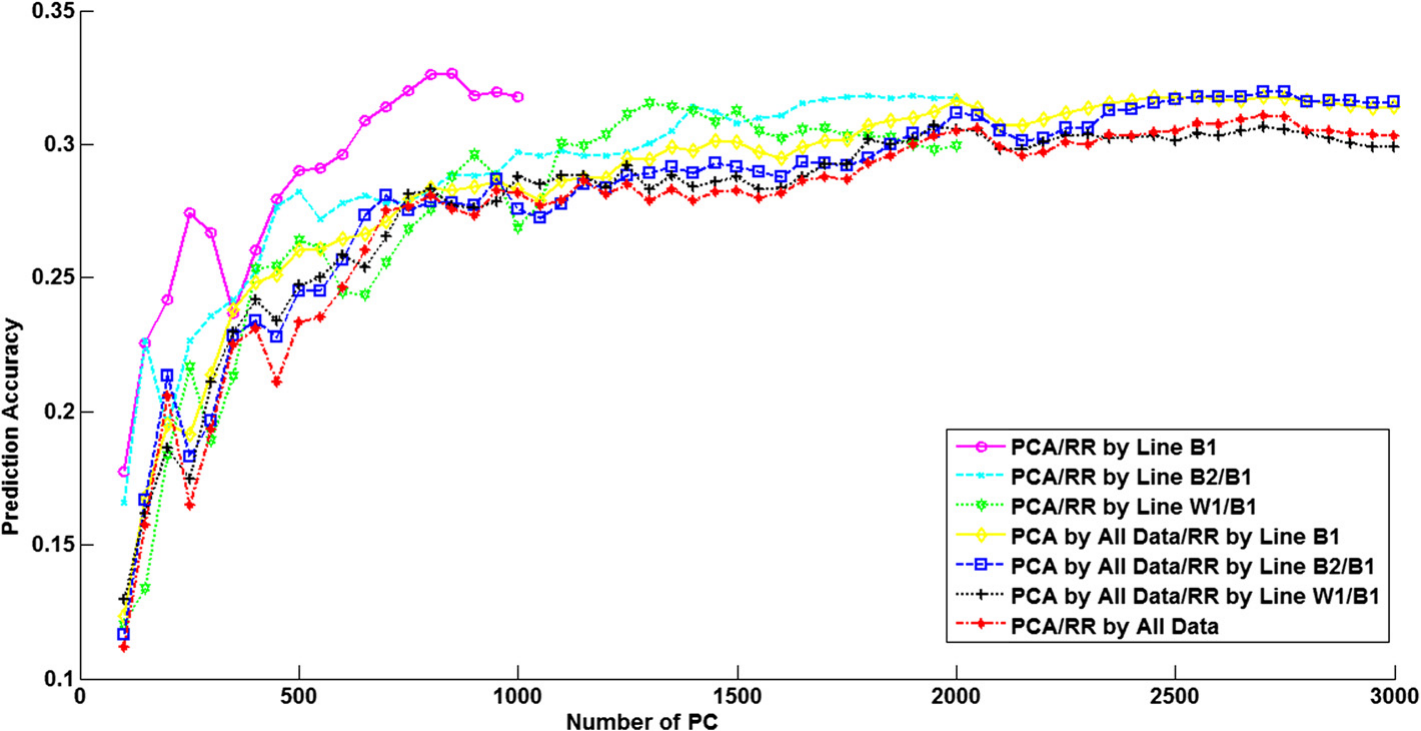
**Relationship between the number of principal components and the prediction accuracy for line B1.** Breeding values for line B1 are predicted using seven different training datasets.

**Figure 4:**
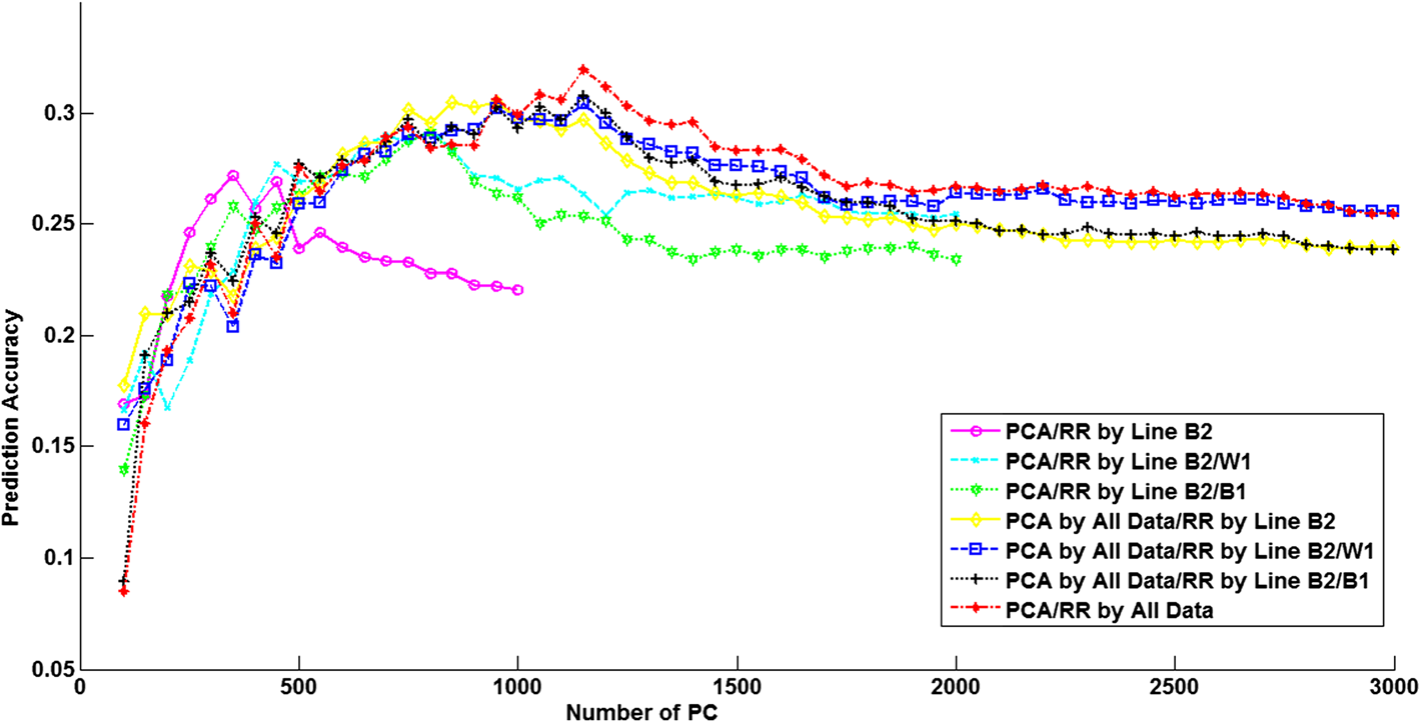
**Relationship between the number of principal components and the prediction accuracy for line B2.** Breeding values for line B2 are predicted using seven different training datasets.

**Figure 5:**
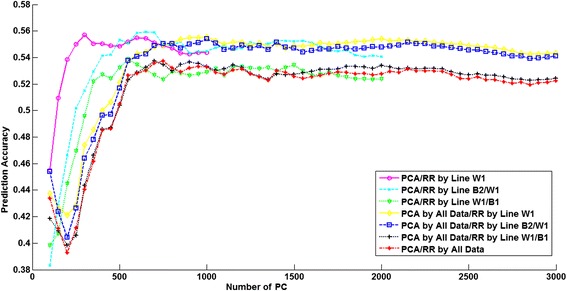
**Relationship between the number of principal components and the prediction accuracy for line W1.** Breeding values for line W1 are predicted using seven different training datasets.

The number of PC that explained 97% of the variance of the genotypes in the training dataset ranged from 452 to 1189 (Table 6). The number of PC clearly increased when another line was added to the training dataset. Prediction accuracies obtained with RRPCA with increasing numbers of PC included in the model are shown in Figures 3, 4 and 5 for the three lines. Here we consider only those scenarios where the training dataset included the evaluated line, i.e. four scenarios per line. The PCA was either based on the training dataset alone or based on all lines, regardless of which lines were included in the training dataset. Note that the number of PC is always smaller than the number of training samples. As a result, the curves in Figures 3, 4 and 5 for scenarios for which PC were derived using only one or two lines in the training dataset did not extend as far across the X-axis as those including all three lines.

**Table 6:** **Number of principal components that account for 97% of the genotypic variance for seven training datasets**

**Training dataset**	**Number of principal components**
B1	515
B2	548
W1	452
B1 + B2	858
B1 + W1	624
B2 + W1	634
B1 + B2 + W1	1189

The results for line B2 shown in Figure 4, clearly indicate that the multi-line training on both PCA and the ridge regression model help to improve the prediction accuracies. Comparing the two scenarios that used the same lines as training data in the ridge regression model but different combinations of lines to perform the PCA shows that the scenario using all lines for PCA always reached a higher maximum accuracy than the scenarios using only the training dataset for PCA. For lines B1 and W1 (Figures 3 and 5), the maximum prediction accuracy was in both cases achieved when only the line itself was used both for PCA and in the ridge regression model, and fewer PC were required to reach this maximum accuracy.

## Discussion

### Gain of multi-line genomic prediction

The main objective of our study was to investigate whether genomic prediction using a training dataset across lines improves the accuracy of genomic prediction within a line, and whether such potential improvement depends on the linear model used. The results showed only a small gain in prediction accuracy for line B2 and no improvement for the other two lines. When using data from the two closely-related lines B1 and B2 together, a consistent small gain in accuracy was observed for line B2 but not for line B1. The difference in the initial accuracies for B1 and B2 likely explains these apparent inconsistent results; line B2 had a relatively lower accuracy when training on its within-line data than line B1. This implies that it was easier for line B2 to gain accuracy from adding information from a closely-related line, while the expected added benefit of adding information from line B2 to line B1 was smaller. Interestingly, using information only from line B1 to predict line B2 resulted in a comparatively smaller prediction accuracy than using only information from line B2 to predict line B1. In any case, the results suggest that, between lines B1 and B2, both the QTL effects and the LD between SNPs and QTL were sufficiently similar to achieve selection response in one line when using SNP effects estimated in the other line. For the unrelated line W1, enlarging the training dataset with the samples from lines B1 and B2 slightly decreased prediction accuracies on average. These results suggest that the QTL effects are too different for line W1 compared to lines B1 and B2, or the LD between SNPs and QTL is not sufficiently conserved across these lines. Based on these findings, we conclude that, for closely-related lines, genomic prediction based on multi-line data that includes data from other lines in addition to data from the line itself, may lead to an increase in prediction accuracy or at least does not substantially degrade the prediction accuracy.

Our findings are in line with the general observation in the literature that the gain from multi-line or multi-breed genomic prediction is at most limited [37–39]. Similar to our study, other groups have also reported that this potential small increase in prediction accuracy is observed when lines or breeds are related [40], which can be evaluated for each trait separately by estimating a genetic correlation between lines or breeds [37]. Moreover, the benefit from including multi-line data tends to be more pronounced for numerically smaller breeds or lines [41], while there is only a very small increase if there is already a lot of information available from the breed or line itself [42]. In our case, the numbers of animals included in the training dataset were very similar across the lines, but as discussed above, the line with the smallest accuracy based on its own data gained most from using a multi-line training dataset.

### Accuracies achieved within lines

The accuracies achieved within each of the three lines clearly differed. With GBLUP, prediction accuracies were equal to 0.50, 0.30 and 0.77 for lines B1, B2 and W1, respectively. The 238 to 240 validation animals had 144, 155 and 186 of their dams included in the training dataset for lines B1, B2 and W1, respectively. This resulted in average squared pedigree-based relationships of 0.0093, 0.0127 and 0.0147 for lines B1, B2 and W1, respectively. Based on these numbers, line B1 was expected to have the lowest accuracy and line W1 the highest [43]. The observed higher accuracy for line B1 compared to B2 was therefore quite unexpected, also considering the observed similarities between these two lines (e.g. Figures 1 and 2) and their similar trait heritabilities. In another study [44], on brown layers with a training dataset of similar size, accuracies of ~0.35 and ~0.5 were reported using GBLUP for early and late egg production, respectively, which falls in the range of accuracies observed here for lines B1 and B2. Line W1 achieved a considerably higher accuracy than lines B1 and B2, because the validation animals for this line had somewhat stronger links with the training animals of the line itself, the trait had a heritability that was ~0.1 higher compared to B1 and B2, and it was more inbred, as commonly observed for white compared to brown layer lines e.g. [45].

### Comparison of models

Across the three lines, no model was consistently better than the others. Remarkably, the Bayesian variable selection models never outperformed the other models, despite our expectation that they can put more weight on SNPs that have consistent LD with QTL across lines. Using the currently available 600k SNP panel [46] instead of the 60k SNP chip is expected to result in at least a small increase in accuracy across all models, and may lead to a relatively larger improvement in accuracy for the Bayesian variable selection models compared to GBLUP. Nevertheless, our results indicate that the investigated trait is polygenic, i.e. that it is controlled by many genes with small effects, which was confirmed in a genome-wide association study that used data from brown layers for a similar trait [47].

The GBLUP and RRBLUP models are expected to be mathematically equivalent [5]. Our results, however, showed correlations between GBLUP_VR and RRBLUP as low as 0.96 for lines B1 and B2 and 0.98 for line W1. In our implementation of the RRBLUP model, the SNP genotypes were centred and scaled, such that the genotype codes for each SNP had a mean of 0 and a variance of 1. The GBLUP_VR model uses a **G** matrix that uses centred genotypes that are not scaled by their variances. Instead of performing the scaling of variances at the level of the genotypes, it is performed for all SNPs simultaneously at the level of the relationships. This implies that the RRBLUP implementation used in our study puts relatively more emphasis on SNPs with low MAF compared to GBLUP_VR, which may explain the observed differences in results between these two models.

Since there are few previous studies on the use of PCA for genomic prediction and because it has been suggested that it may be particularly beneficial for across population genomic prediction [27], we analyzed its potential impact on prediction accuracy. If we compare RRBLUP and RRPCA, the only model difference is whether PCA is applied or not. In line B2, RRPCA performed clearly better than RRBLUP. Extracting the PC using the training dataset of all lines instead of using only the line itself may lead to somewhat higher accuracies, as suggested by Figures 3, 4 and 5. Results showed that for lines B2 and W1 (Figures 4 and 5), the maximum accuracy was reached when using only the first ~40% of the PC, which accounted for 95% of the variance in the genotype data. This suggests that models that internally perform variable selection, such as BayesC, are expected to be better able to put most emphasis on those genotypes that are important, conditional on the observed phenotypes. However, this was not confirmed, since BayesC was generally among the models with the lowest accuracy. As discussed previously, this may be alleviated by using a higher SNP density. Another reason that may explain why BayesC did not outperform the other models could be that a suboptimal value of the parameter π was used; the number of PC used increased up to twofold when training animals of all three lines were used (Table 6), and therefore an implementation of e.g. BayesC that explicitly estimates π [7] may achieve higher accuracies. In any case, the RRPCA results suggest that regularization by minimizing the *L*_2_-norm of linear weights is not sufficient to alleviate the over-fitting problem of genomic prediction. The impact of this over-fitting may be much more pronounced when the number of SNPs increases drastically compared to the number of phenotypes, as would be the case when using whole-genome sequence data.

### Bias of genomic predictions

The regression coefficients (see Additional file 1: Tables S1, S2 and S3) showed that substantial bias was present in several scenarios. However, across training datasets that included the evaluated line, the regression coefficients were generally relatively consistent within models. Regression coefficients for genomic predictions using only the line itself as training data were similar to those for EBV from pedigree-based BLUP. Standard errors of the regression coefficients were high but lowest when the evaluated line was included in the training dataset. The standard errors indicated that the regression coefficients were not significantly different from 1 for lines B1 and B2, across nearly all combinations of models and training datasets, (see Additional file 1: Tables S1 and S2). However, for line W1, the regression coefficients were in nearly all cases significantly different from 1 (see Additional file 1: Table S3). The regression coefficients suggested that the variance of the EBV tended to be underestimated for lines B1 and W1, perhaps because the available animals with genotype and phenotype information in the training data for each line did not cover the whole range of selection candidates, i.e. only ~2/3 of the validation animals had their dam included in the training dataset for these two lines. However, for line B2 also only ~2/3 of the validation animals had their dam included in the training dataset, and for this line the variance of the EBV was across models generally slightly overestimated. In some scenarios for lines B1 and W1, when the evaluated line was not included in the training dataset, the regression coefficients had substantial negative values. Combined with the substantial negative accuracies, which were significantly lower than 0 for line W1, this suggests that some QTL have opposite phase with the surrounding SNPs in the different lines. It is, however, quite unlikely that this is the case for most of the QTL, which is required to explain the negative accuracies. Therefore, it remains unclear what the cause of the negative accuracies and regression coefficients is.

### Restrictions of linear models for multi-line training

Small improvements in the accuracy of genomic predictions from using the multi-line training datasets were observed for line B2 but not for lines B1 and W1. Data heterogeneity might be one of the major reasons for these line differences, i.e. the allele frequencies differed between lines. However, linear models estimate the average marker effects and maximize the prediction performance over the whole training dataset, which means that estimates obtained from multi-line training do not necessarily best fit the data from each line. Therefore, another promising direction of multi-line genomic prediction may be to model the data locally, rather than across the whole training dataset. Effectively, such models would be able to put the greatest emphasis on information from closely-related individuals, while effectively ignoring information from distantly-related individuals. Thus, such models do not make assumptions about linearity across the whole data and would be non-linear by nature. The performance of non-linear models for multi-line genomic prediction is investigated in a follow-up study.

## Conclusions

Our results indicate that multi-line genomic prediction may be effective when lines are closely-related. In the case of multi-line training with two distantly-related chicken lines, genomic prediction using only the line itself yielded similar or slightly lower accuracies than multi-line genomic prediction. Bayesian variable selection models and GBLUP type of models generally gave similar accuracies. The RRPCA model yielded substantially higher accuracies for one line, which suggests that using a compact representation of the genotype data, as achieved by PCA, can indeed alleviate the severe “*n* < *p*” problem in genomic prediction, although this appears to be line-specific. The Bayesian variable selection models were also expected to be able to achieve such selective representation of the genotype data, but were not able to outperform the other models. Performance of the Bayesian models could perhaps be enhanced by using higher density data, or by allowing the proportion of selected markers (1-π) to be determined by the model.

## Additional file

Additional file 1: Table S1. Coefficients of regression of observed phenotypes on predicted breeding values of seven linear methods in seven training scenarios for line B1. Description: BLUP: conventional BLUP using a pedigree based relationship matrix; G-BLUP: Genome-enabled Best Linear Unbiased Prediction (G-BLUP); RRBLUP: Ridge Regression BLUP; RRPCA: Ridge Regression with PCA reduction; BayesSSVS: Bayesian Stochastic Search Variable Selection; BayesC. **Table S2.** Coefficients of regression of observed phenotypes on predicted breeding values of seven linear methods in seven training scenarios for line B2. BLUP: conventional BLUP using a pedigree based relationship matrix; G-BLUP: Genome-enabled Best Linear Unbiased Prediction (G-BLUP); RRBLUP: Ridge Regression BLUP; RRPCA: Ridge Regression with PCA reduction; BayesSSVS: Bayesian Stochastic Search Variable Selection; BayesC. **Table S3.** Coefficients of regression of observed phenotypes on predicted breeding values of seven linear methods in seven training scenarios for line W1. BLUP: conventional BLUP using a pedigree based relationship matrix; G-BLUP: Genome-enabled Best Linear Unbiased Prediction (G-BLUP); RRBLUP: Ridge Regression BLUP; RRPCA: Ridge Regression with PCA reduction; BayesSSVS: Bayesian Stochastic Search Variable Selection; BayesC.
