# Supplementary material for: Genomic prediction based on data from three layer lines: a comparison between linear methods
Source: Genet Sel Evol. 2014 Oct 1;46(1):57. doi: 10.1186/s12711-014-0057-5 (PMC4180920; doi:10.1186/s12711-014-0057-5)
Supplement: Additional file 1: Table S1. — Coefficients of regression of observed phenotypes on predicted breeding values of seven linear methods in seven training scenarios for line B1. Description: BLUP: conventional BLUP using a pedigree based relationship matrix; G-BLUP: Genome-enabled Best Linear Unbiased Prediction (G-BLUP); RRBLUP: Ridge Regression BLUP; RRPCA: Ridge Regression with PCA reduction; BayesSSVS: Bayesian Stochastic Search Variable Selection; BayesC. Table S2. Coefficients of regression of observed phenotypes on predicted breeding values of seven linear methods in seven training scenarios for line B2. BLUP: conventional BLUP using a pedigree based relationship matrix; G-BLUP: Genome-enabled Best Linear Unbiased Prediction (G-BLUP); RRBLUP: Ridge Regression BLUP; RRPCA: Ridge Regression with PCA reduction; BayesSSVS: Bayesian Stochastic Search Variable Selection; BayesC. Table S3. Coefficients of regression of observed phenotypes on predicted breeding values of seven linear methods in seven training scenarios for line W1. BLUP: conventional BLUP using a pedigree based relationship matrix; G-BLUP: Genome-enabled Best Linear Unbiased Prediction (G-BLUP); RRBLUP: Ridge Regression BLUP; RRPCA: Ridge Regression with PCA reduction; BayesSSVS: Bayesian Stochastic Search Variable Selection; BayesC. [file 12711_2014_57_MOESM1_ESM.docx]

**Additional file 1**

**Table S1 – Coefficients of regression of observed phenotypes on predicted breeding values of seven linear methods in seven training scenarios for line B1**

|  | Training data | | | | | | |
| --- | --- | --- | --- | --- | --- | --- | --- |
| Model | B1 | B2 | W1 | B1+B2 | B1+W1 | B2+W1 | B1+B2+W1 |
| BLUP^1^ | 1.385 | - | - | - | - | - | - |
| GBLUP_VR | 1.231 | 0.958 | -0.531 | 1.099 | 1.160 | 0.717 | 1.040 |
| GBLUP_%id | 1.662* | 1.507 | -1.058 | 1.529 | 1.170 | 0.721 | 1.478 |
| RRBLUP | 1.467 | 1.480 | -0.033 | 1.418 | 1.396 | 1.189 | 1.343 |
| RRPCA | 1.288 | 1.000 | 1.218 | 1.167 | 1.242 | 0.964 | 1.129 |
| BSSVS | 1.368 | 1.037 | -1.529 | 1.216 | 1.390 | 0.893 | 1.227 |
| BayesC | 1.350 | 1.018 | -1.393 | 1.209 | 1.383 | 0.879 | 1.225 |
| Min SE^1^ | 0.216 | 0.366 | 0.918 | 0.216 | 0.207 | 0.319 | 0.208 |
| Max SE^1^ | 0.293 | 0.518 | 1.418 | 0.287 | 0.295 | 0.454 | 0.280 |

^1^Minimum and maximum SE across the genomic prediction models.

*Value is significantly greater than 1.0.

**Table S2 – Coefficients of regression of observed phenotypes on predicted breeding values of seven linear methods in seven training scenarios for line B2**

|  | Training data | | | | | | |
| --- | --- | --- | --- | --- | --- | --- | --- |
| Model | B1 | B2 | W1 | B1+B2 | B1+W1 | B2+W1 | B1+B2+W1 |
| BLUP^1^ | - | 0.800 | - | - | - | - | - |
| GBLUP_VR | 0.383* | 0.696 | 0.893 | 0.623 | 0.501 | 0.752 | 0.701 |
| GBLUP_%id | 0.644 | 0.981 | 1.407 | 0.882 | 0.806 | 1.011 | 0.962 |
| RRBLUP | 0.587 | 1.036 | 0.977 | 1.044 | 0.716 | 1.008 | 1.039 |
| RRPCA | 0.594 | 1.153 | 0.966 | 1.188 | 0.675 | 1.149 | 1.195 |
| BSSVS | 0.468 | 0.803 | 1.081 | 0.767 | 0.594 | 0.883 | 0.847 |
| BayesC | 0.458 | 0.797 | 1.149 | 0.763 | 0.523 | 0.900 | 0.840 |
| Min SE^1^ | 0.306 | 0.217 | 0.689 | 0.199 | 0.285 | 0.207 | 0.194 |
| Max SE^1^ | 0.430 | 0.279 | 1.043 | 0.267 | 0.394 | 0.262 | 0.253 |

^1^Minimum and maximum SE across the genomic prediction models.

*Value is significantly smaller than 1.0.

BLUP: conventional BLUP using a pedigree based relationship matrix; G-BLUP: Genome-enabled Best Linear Unbiased Prediction (G-BLUP); RRBLUP: Ridge Regression BLUP; RRPCA: Ridge Regression with PCA reduction; BayesSSVS: Bayesian Stochastic Search Variable Selection; BayesC.

**Table S3 – Coefficients of regression of observed phenotypes on predicted breeding values of seven linear methods in seven training scenarios for line W1**

|  | Training data | | | | | | |
| --- | --- | --- | --- | --- | --- | --- | --- |
| Model | B1^1^ | B2^1^ | W1^2^ | B1+B2^1^ | B1+W1^2^ | B2+W1^2^ | B1+B2+W1^2^ |
| BLUP^1^ | - | - | 1.429 | - | - | - | - |
| GBLUP_VR | -3.147 | -1.754 | 1.273 | -3.033 | 1.270 | 1.312 | 1.325 |
| GBLUP_%id | -4.635 | -2.261 | 1.547 | -4.366 | 1.556 | 1.577 | 1.604 |
| RRBLUP | -3.953 | -3.173 | 1.225 | -5.499 | 1.465 | 1.530 | 1.544 |
| RRPCA | -3.068 | -2.951 | 1.395 | -3.133 | 1.353 | 1.448 | 1.405 |
| BSSVS | -2.963 | -2.901 | 1.507 | -3.684 | 1.438 | 1.483 | 1.443 |
| BayesC | -2.950 | -2.864 | 1.524 | -3.602 | 1.446 | 1.486 | 1.444 |
| Min SE^3^ | 0.822 | 1.016 | 0.124 | 0.678 | 0.136 | 0.134 | 0.140 |
| Max SE^3^ | 1.482 | 1.521 | 0.152 | 1.227 | 0.162 | 0.158 | 0.167 |

^1^Across all models, (almost) all regression coefficients were significantly smaller than 1.0.

^2^Across all models, (almost) all regression coefficients were significantly greater than 1.0.

^3^Minimum and maximum SE across the genomic prediction models.

BLUP: conventional BLUP using a pedigree based relationship matrix; G-BLUP: Genome-enabled Best Linear Unbiased Prediction (G-BLUP); RRBLUP: Ridge Regression BLUP; RRPCA: Ridge Regression with PCA reduction; BayesSSVS: Bayesian Stochastic Search Variable Selection; BayesC.
